# Comprehensive improvement of induced ruminal acidosis in lambs: targeted microencapsulated essential oils and marine algae restore rumen function, systemic health, immunity, and welfare

**DOI:** 10.3389/fvets.2026.1877156

**Published:** 2026-09-01

**Authors:** Asmaa T. Y. Kishawy, Eman Metwally, Ioan Pet, Ghadeer A. Attia, Fayza A. Ahmed, Hala Saed, Heba Moharam, Asmaa S. Mohammed, Mirela Ahmadi, Gabi Dumitrescu, Zainab F. Helal, Ahmed Abdelfattah-Hassan, Sherief Mohamed Abdel-Raheem, Doaa Ibrahim

**Affiliations:** 1 Department of Nutrition and Clinical Nutrition, Faculty of Veterinary Medicine, Zagazig University, Zagazig, Egypt; 2 Faculty of Veterinary Medicine, Veterinary Teaching Hospital, Zagazig University, Zagazig, Egypt; 3 Department of Biotechnology, Faculty of Bioengineering of Animals Resources, University of Life Sciences “King Mihai I” from Timisoara, Timisoara, Romania; 4 Department of Behaviour and Management of Animal, Poultry and Aquatic, Faculty of Veterinary Medicine, Zagazig University, Zagazig, Egypt; 5 Department of Internal Medicine and Infectious Diseases, Faculty of Veterinary Medicine, Veterinary Teaching Hospital, Mansoura University, Mansoura, Egypt; 6 Department of Behaviour and Animal Management, Faculty of Veterinary Medicine, Mansoura University, Mansoura, Egypt; 7 Department of Pharmacology, Zagazig Scientific and Medical Research Center (ZSMRC), Zagazig University, Zagazig, Egypt; 8 Department of Veterinary Medicine, College of Applied and Health Sciences, A'Sharqiyah University, Ibra, Oman; 9 Department of Anatomy and Embryology, Faculty of Veterinary Medicine, Zagazig University, Zagazig, Egypt; 10 Department of Public Health, College of Veterinary Medicine, King Faisal University, Al-Ahsa, Saudi Arabia

**Keywords:** behavior, induced ruminal acidosis, inflammatory markers, marine calcareous algae, microencapsulated essential oils, rumen fermentation, total volatile fatty acid

## Abstract

This investigation was performed to evaluate the efficacy of a microencapsulated essential oils (MEOs) blend and marine calcareous algae (Marine Cal-Algae) to counteract experimentally induced ruminal acidosis (RA) in lambs by evaluating animal performance, hematological indices, general health condition, inflammatory markers, rumen fermentation milieu, and animal behavior. This study was performed on 45 yearling rams weighing 30–35 kg, randomly assigned to five experimental groups (nine lambs/group with three replicates for each), as follows: a control (healthy lambs) group and four induced RA groups. These included an untreated RA group and three treated groups receiving either essential oils (MEOs), marine calcareous algae, or a combination of both treatments. All treatment regimens lasted for 6 days after RA induction. The results revealed that skin turgor, blood capillary refill time, respiratory and pulse rates were significantly increased, while ruminal motility was significantly reduced after RA induction. Notably, the combined MEOs–marine calcareous algae treatment restored these parameters, markedly lowering skin turgor, capillary refill time, and respiratory and pulse rates while improving ruminal motility to near-control values. In addition, RA reduced lysozyme and myeloperoxidase (MPO) values, while nitric oxide (NO), C-reactive protein (CRP), and interleukin-6 (IL-6) were significantly increased. Conversely, treatment with both MEOs and marine calcareous algae mitigated these effects. Lactic acidosis decreased rumen pH, protozoa activity, and acetate:propionate ratio but increased lactate and total volatile fatty acids (TVFAs), while dual MEO-marine algae treatment reversed these shifts. Stress responses in the open field test (OFT) escalated post-acidosis (↑escapes, beats, latency; ↓exploratory sniffing), indicating heightened anxiety that was substantially attenuated by MEOs-marine algae intervention. Therefore, MEOs and marine calcareous algae supplementation can help veterinarians treat lambs with RA and promote faster recovery through natural interventions.

## 1 Introduction

Ruminal acidosis (RA) is a prevalent metabolic disorder in ruminants, including sheep, characterized by repeated episodes of moderately depressed ruminal pH, typically ranging between 5.2 and 5.8 (1). RA is primarily associated with the consumption of high-concentrate, low-fiber diets that promote rapid fermentation of carbohydrates in the rumen. This process leads to excessive production of volatile fatty acids (VFAs) and lactic acid, overwhelming the rumen's buffering capacity, mainly provided by saliva and rumination. As a result, the ruminal pH declines, disrupting the microbial ecosystem and impairing normal digestive functions (2). In sheep, RA has been linked to reduced feed intake, decreased nutrient digestibility, impaired growth performance, and alterations in rumen microbial populations. Additionally, it may contribute to secondary complications such as rumenitis, muscle weakening, cardiovascular collapse, hemoconcentration, renal failure, liver abscesses, laminitis, and systemic inflammation due to the translocation of endotoxins into the bloodstream (3). These effects collectively result in significant economic losses in sheep production systems. Essential oils (EOs) are complex blends of bioactive secondary metabolites, predominantly terpenoids and phenylpropanoids, known to beneficially modulate ruminal fermentation and enhance nutrient utilization in ruminants (4). Beyond their nutritional role, EOs have attracted considerable attention for their potent antimicrobial properties and are increasingly explored as natural rumen modifiers (5). Their bioactivity stems from their ability to disrupt the cellular integrity and metabolic functions of bacteria, fungi, and protozoa, thereby exhibiting efficacy comparable to conventional therapeutic approaches (6). In addition, essential oils have been shown to reduce oxidative stress and exert immunomodulatory effects (7). Their antimicrobial activity is primarily linked to their hydrophobic properties, which allow them to interact with and disrupt microbial cell membranes, increasing permeability and leading to cellular dysfunction (8). Such interactions can suppress key ruminal processes, including protein deamination and methanogenesis (9). As a result, nitrogen metabolism is altered, characterized by reduced ruminal ammonia, methane, and acetate concentrations, together with enhanced propionate and butyrate production (10). Essential oils exhibit ionophore-like properties that can modulate gastrointestinal development and ruminal microbial ecology, ultimately enhancing feed efficiency and reducing neonatal disorders (4, 9). These bioactivities are associated with a shift in rumen fermentation toward reduced acetate production and increased propionate and butyrate proportions, which contributes to improved feed utilization and lower methane (CH_4_) emissions (11). Furthermore, certain essential oils selectively suppress *Veillonellaceae* populations (12), a microbial group inversely related to total volatile fatty acid (TVFA) production and average daily gain (ADG) in sheep (13). Oregano essential oil has been reported to increase ruminal pH and inhibit lactic acid-producing bacteria, thereby preventing the onset of acidosis under high-concentrate feeding conditions. Additionally, it can shift fermentation toward increased propionate production and reduced acetate levels, improving energy utilization and reducing methane emissions (14). Cinnamon essential oil, which is high in carvacrol and cinnamon aldehyde, possesses strong antioxidant and antibacterial properties and may improve animal performance (15). Clove essential oil, primarily composed of the active compound eugenol, has gained attention as a natural phytogenic additive for modulating rumen fermentation and mitigating metabolic disorders such as ruminal acidosis (RA). Studies have shown that Clove essential oil can modulate rumen microbial communities, enhance beneficial microbes while suppressing harmful or excessive fermenters. This microbial shift improves fermentation efficiency. It prevents excessive production and accumulation of volatile fatty acids (VFAs) and lactic acid, both major contributors to ruminal pH decline in RA (16, 17). However, the practical application of essential oils in ruminant nutrition remains constrained by their inherent instability, susceptibility to environmental factors, and rapid degradation within the rumen (18). To overcome these limitations, encapsulation technologies have been developed to preserve their bioactive constituents, improve stability, and enable controlled, targeted release (19). Microencapsulation is a protective strategy that isolates core bioactive compounds from oxygen, light, and moisture while minimizing interactions with surrounding matrices. Applied to essential oils, this approach enhances physicochemical stability, prolongs shelf life, and improves their delivery and bioavailability in the rumen (20). Calcareous marine algae (CMA), particularly *Lithothamnium calcareum*, have emerged as promising natural buffering agents in ruminant nutrition due to their high content of bioavailable calcium carbonate and trace minerals (21). These algae function as slow-release rumen buffers, helping to neutralize excess acidity in the rumen environment. One of the primary mechanisms by which CMA mitigates RA is through its buffering capacity, which stabilizes ruminal pH and reduces the duration of acidotic episodes. Additionally, CMA supports the growth of fiber-digesting bacteria and prevents excessive proliferation of acid-producing microbes, which improves rumination behavior and feeding patterns (22). The combined use of microencapsulated essential oils (MEOs) and marine calcareous algae (Marine Cal-Algae) offers a synergistic nutritional strategy to manage sub-acute ruminal acidosis (RA) in lambs by integrating microbial modulation by MEOs synergistically with physicochemical buffering of CMA. This study aims to evaluate the synergistic effect of microencapsulated essential oils and calcareous marine algae on the mitigation of acute ruminal acidosis (RA) in sheep by assessing their influence on ruminal pH stability, fermentation characteristics, inflammatory markers, and overall animal health under high-concentrate feeding conditions.

## 2 Materials and methods

### 2.1 Ethical statement

Institutional Review Board Statement: the experimental protocols and exposure techniques were approved by the Institutional Fish Use and Care Committee of the Faculty of Veterinary Medicine, Zagazig University Ethics Committee (ZUIACUC/2/F/236/2025).

### 2.2 Experimental animals and treatment protocol

**Figure 1:**
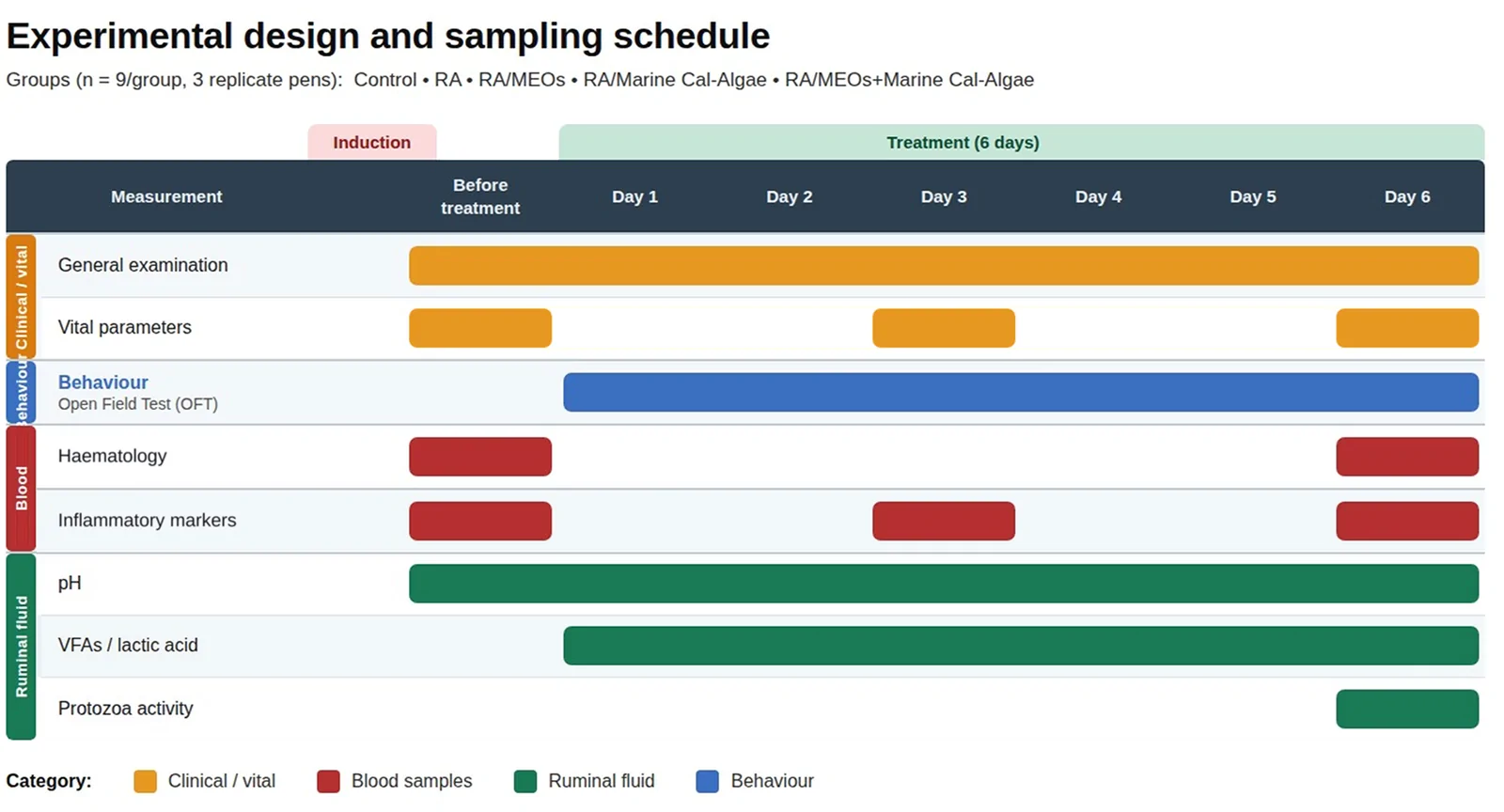
Sampling schedule for experimental design.

The current study was carried out at the experimental section of farm animals at the Faculty of Veterinary Medicine, Zagazig University, Egypt. A total of 45 animals were randomly assigned to five experimental groups (nine rams/group with three replicates per group), with body weights ranging from 30 to 35 kg. On admission, a thorough physical examination was done for all animals to ascertain their health status. After that, all animals were housed in replicates, fed *ad libitum* on a forage-based diet and water, and kept on wheat straw bedding. The lambs were fed twice per day—at 8.00 a.m. and at 4.00 p.m.—and were provided with a mineral mixture *ad libitum*. This experimental study was done in two stages after a 21-day acclimatization period. Lambs were off-fed for 24 h before the start of the clinical. The first stage included experimental induction of ruminal acidosis (RA) in rams via feeding 50 g/kg body weight/head of wheat flour at 0 and 12 h for two consecutive days. The flour was delivered as a warm aqueous suspension using a gastric tube. The second stage included the use of a microencapsulated essential oil mixture, calcareous marine algae, or both as additives for 6 days after acidosis induction. The post-induction (recovery) ration was therefore an all-forage diet with a forage-to-concentrate ratio of 100:0, offered at 1.3 kg DM/head/day; no concentrate was fed at any point after acidosis induction, so that the ruminal fermentation responses recorded during the treatment period reflect the additives rather than a continuing dietary starch load. The experimental lambs were divided randomly into five groups as follows: control group (which includes healthy lambs without induction of ruminal acidosis) and four ruminal acidosis-induced groups); RA group (which includes lambs that afflicted by experimentally induced ruminal acidosis without treatment), RA/MEOs group (which includes lambs that afflicted by experimentally induced ruminal acidosis and treated with treatment essential oils mix), RA/Marine Cal-Algae group (which include lambs afflicted by experimentally induced ruminal acidosis and treated with marine calcareous algae), and RA/MEOs +Marine Cal-Algae group (which includes lambs that afflicted by experimentally induced ruminal acidosis and treated with MEOs +Marine Cal-Algae). Essential oils comprising cinnamon, cloves, oregano, and peppermint were obtained from Flow Actives company, San Donato Milanese, which comprises (cinnamon; FL 010: cloves; FL 026: oregano; FL 031 and peppermint; FL 001). The microencapsulation matrix (particle size 100–600 μm) was chosen to protect the volatile actives and to favor controlled release and retention within the ruminal raft. The essential oils mix was prepared by blending these individual microencapsulated oils at a ratio of 1:1:1:1 at a dose of 2 g/kg dry matter. The manufacturer reports a ruminal release of >80% of the encapsulated actives over 12 h *in situ*, with a corresponding rumen-bypass fraction of < 20%; the twice-daily gavage schedule was adopted to maintain ruminal exposure across the whole 24-h cycle. This dose was selected in accordance with the manufacturer's technical recommendation for the microencapsulated blend and falls within the range of essential-oil inclusion rates previously reported to modulate ruminal fermentation and stabilize ruminal pH in small ruminants without depressing feed intake (4, 6, 16). Marine calcareous algae were purchased from Celtic Sea Minerals (Carrigaline, P43 NN62, Ireland, Acid Buf sheep with Ca 300 g/kg; Mg 55 g/kg; ash 950 g/kg) at the dose of 11 g/kg dry matter, following the manufacturer's recommendation, with a twice-daily gavage schedule orally twice daily for 6 days. The sampling schedule was illustrated in Figure 1. This inclusion rate corresponds to the manufacturer's recommended buffering dose for the calcareous marine-algae product and is consistent with the effective doses of Lithothamnium-based buffers reported to raise ruminal pH and mitigate acidosis in ruminants (21).

### 2.3 Clinical observation

#### 2.3.1 General examination

Experimental ruminal acidosis-induced lambs were examined daily during the experimental period; the posture, anorexia, recumbency, hunger, abdominal distension, rumination, watery diarrhea, and grinding of teeth of each group were observed. General examination was performed both prior to and following therapy, according to the method described by Abdela (23).

#### 2.3.2 Vital parameters (pulse, temperature, respiration, and capillary refill time)

The vital parameters were measured before RA treatment and after 3 and 6 days of RA treatment. The pulse rate was taken at the femoral artery, gently compressed with the fingers until the waves were identified. The motions of the abdomen and rib cage were numbered to determine the respiration rate. A digital clinical thermometer was used to take the body temperature via the rectum through gentle insertion for 2 min. The degree of dehydration in sheep with RA was assessed by measuring the blood capillary refill time using gum pressure and the tip of the finger. Ruminal motility and skin turgor were also noted. The aforementioned parameters are estimated as described by Han et al. (24).

### 2.4 Behavioral evaluations

The open field test (OFT) was utilized to evaluate social isolation and behavioral reactions to a new object. Every lamb was put in a different arena after RA induction for six consecutive days during treatment. The arena measured 1 m × 1.5 m with 2.5 m walls, was uniformly illuminated at 250 lux, and was placed at the center of the arena; testing was conducted at a fixed time after the morning meal. Each session lasted 2 h following the morning meal. Two video cameras mounted on a wooden panel around the arena were used to record behavior for 20 min during the measurement period. Each animal's hind legs entering the wide field marked the start of the observation session. Walking, lying, eating, drinking, licking salt, standing awake, and standing passively were among the states seen. Greiveldinger et al. (25) characterized standing awake as an immobile position with the head and ears looking upward, whereas standing inactively corresponded to an immobile position with the head and ears pointing downward. The following behavioral types were noted: “latency to sniff the novel object for the first time,” which gauges how long it takes a sheep to make contact with the object using its nose; “number of bleats,” which counts the number of times the sheep's nose made contact with the object; “sniffing of the ground or wall,” which reflects the instances of nasal contact with the ground or arena walls; and “escape attempts,” which describes the actions of sheep trying to jump or collide with the walls in an attempt to leave the open field. To provide uniform testing conditions, the floor was cleansed after every test, as performed previously by Qianrige et al. (26).

### 2.5 Hematological examination

A 5-mL blood sample was collected from RA acidotic lambs' jugular vein (before treatment of RA and 6 days post treatment, *n* = 6/group) using tubes containing ethylene diamine tetra acetic acid (EDTA). An automatic hematology analyzer was used to measure total white blood cell (WBC) count, total red blood cell (RBC) count, hemoglobin estimation (Hb%), and packed cell volume (PCV%). An automated hematology analyzer was used to perform the hematological examination as the method previously mentioned by Jain (27).

### 2.6 Biochemical examination

#### 2.6.1 Inflammatory markers

The extracted serum that was collected from RA-acidotic lambs' veins (before treatment of RA and after 3 and 6 days of RA treatment, *n* = 6/group) was used for measuring lysozymes, C-reactive protein (CRP), interleukin-6 (IL-6), nitric oxide (NO), and myeloperoxidase (MPO) activities before and after treatment. According to Engstad et al. (28), the turbidimetric approach was used to quantify the serum lysozyme levels. The turbidimetric assay (Spectrum Diagnostics; Egypt) was used to quantify the amounts of serum CRP in accordance with the procedure outlined by Gewurz et al. (29). Fast-response amperometric electrode sensors according to Zhang and Broderick (30) can be used to quantify NO. The enzyme-linked immunosorbent assay (ELISA) kits (IL-6: ml023756 Shanghai Enzyme-linked Biotechnology Co., Ltd., Minhang, Shanghai, China) were also used to measure serum IL-6 concentrations. According to Quade and Roth, MPO activity was assessed. The technique uses 3,3′,5,5′-tetramethylbenzidine hydrochloride as a sensitive peroxidase substrate for MPO-H_2_O_2_ oxidation (31).

### 2.7 Ruminal fluid analysis

#### 2.7.1 Rumen liquor pH

Rumenocentesis was performed using a disposable syringe and 16-gauge needles to collect ruminal fluid from RA acidotic lambs (before treatment of RA and 6 days during treatment; 2 h after the morning feeding). The ventral rumen on the left side, five centimeters behind the last rib, was the penetration site (32). Ruminal pH was assessed using the ruminal fluid and quantified using a pH meter or pH indicator paper (33). After placing the ruminal fluid on the paper, the pH was measured by comparing the litmus paper's color to the indicator paper's standard colors. After placing the ruminal fluid in a beaker, a pH meter was put inside. Therefore, the mean value was used to evaluate the pH value and measured according to Jaramillo-López et al. (34).

#### 2.7.2 Lactic and total volatile fatty acids

Oral stomach tubing was used to collect 100 mL of ruminal fluid from each RA acidotic lambs' 2 h after the morning feeding (at six consecutive days during treatment) to capture the majority of the noticeable effects of the feeding treatment on the ruminal environment and, consequently, on behavior. Before centrifugation, the obtained rumen fluid samples were stored in an ice box after being filtered through double-layered medical gauze. To eliminate solid particles, all samples were centrifuged for 4 min at 10,000 *g*. After that, the samples were kept at −80 °C for additional examination. To measure the volatile fatty acid (VFA) content, ruminal fluid was maintained by adding 1 mL of metaphosphoric acid (25% wt/vol). Thawed rumen fluid samples were centrifuged for 15 min at 10,000 × g at 4 °C to determine VFA. After adding 200 μL of 1% wt/vol crotonic acid to 2 mL of the supernatant, the mixture was filtered through a 0.45 μm filter. Using a 30 m × 0.32 mm × 0.33 μm fused silica column (DB-FFAP, Agilent Technologies, USA) and a gas chromatograph (Trace 1300, Thermo Fisher Scientific, USA), as reported by Li et al. (35). Lactic acid was analyzed according to Snyder (36).

#### 2.7.3 Protozoa activity test

A coverslip was placed over a drop of ruminal fluid from each RA acidotic lamb (at 6 days post-treatment, *n* = 6/group) on a glass slide. Next, a low-power microscope was used to check for ruminal protozoa. Four groups were used to assess the motility of protozoa: more than 10 movable protozoa per field are classified as highly motile (4); moderate (3): 6–9 movable protozoa per field; mild (2): 3–5 motile protozoa per field are classified as retarded movability; absent (1): no or sporadic alive fauna (very low): < 3 motile protozoa per field, as mentioned in the method Abebaw et al. (37).

### 2.8 Remedial effectiveness of various therapeutic regimens

Therapeutic effectiveness was evaluated daily for each lamb inside the replicate throughout the 6-day treatment period using three pre-defined criteria, applied simultaneously. The clinical recovery data are cumulative counts and proportions of recovered lambs rather than continuous variables and were therefore analyzed by methods appropriate to binary and time-to-event outcomes. The healthy control group was not included in these comparisons because it was not challenged and therefore had no recovery event to record. All analyses were performed on the individual-animal data of the six lambs per group. (i) Resolution of clinical signs: return of appetite and rumination, disappearance of watery diarrhea, abdominal distension and teeth grinding, and a normal standing posture with a willingness to move. (ii) Normalization of vital parameters: rectal temperature 38.5–39.5 °C, pulse rate < 90 beats/min, respiratory rate < 40 breaths/min, capillary refill time ≤ 2 s, skin tent time ≤ 2 s and ruminal motility ≥3 contractions/3 min, i.e., all within the reference intervals defined by the healthy control group. (iii) Restoration of rumen function: ruminal pH ≥ 5.8 with visible protozoal motility on direct microscopy. A lamb was scored as “recovered” on the first day on which all three criteria were met simultaneously and remained met thereafter (38).

### 2.9 Statistical analysis

Data were analyzed using a statistical model appropriate to the experimental design, with the replicate pens as the experimental unit. In this study, after normalizing the distribution using log transformation, one-way analysis of variance (ANOVA) was performed to confirm the effects of single-time-point outcomes. For analysis of clinical signs and immune-related markers, a multivariate general linear model (GLM, two-way ANOVA) was used, with two fixed factors, treatment (five groups) and time (before treatment, after 3 days, after 6 days), and their interaction. For the repeatedly measured outcomes, a linear mixed-effects/repeated-measures model was used. In the repeated-measures model, treatment group, sampling time (day), and their treatment × time interaction were included as fixed effects, while the animals nested within pen (replicate) were specified as a random (subject) effect to account for the repeated measurements collected on the same animal across time points. Behavioral variables from the OFT were log-transformed prior to analysis. Time to clinical recovery (Table 1) was analyzed as time-to-event data at the animal level, using the log-rank test for overall and pairwise comparisons and the Freeman–Halton extension of Fisher's exact test for the proportion recovered on each individual day. Tukey-adjusted *post hoc* comparisons were used for multiple comparisons of significant main effects and interactions. The recovery data in Table 1 are summarized at the pen/group level and were not treated as independent per-animal replicates. Values (means ± standard error of the mean [SEM]) were considered significantly different from the control healthy one when *p* ≤ 0.05.

**Table 1:** Efficacy of combined therapeutic aids comprising microencapsulated essential oils and Marine Cal-Algae on hematological indices in lambs afflicted by experimentally induced ruminal acidosis.

Hematological indices	Experimental group					SEM	*p*-Value
	Control	RA	RA/MEOs	RA/Marine Cal-Algae	RA/MEOs+Marine Cal-Algae		
RBCs × 10^6^ μL							
Before treatment	11.48	11.61	11.53	11.41	11.50	0.10	0.984
After treatment	11.49	11.76	11.58	11.83	11.59	0.09	0.764
WBCs × 10^3^ μL							
Before treatment	5.86^b^	11.55^a^	11.85^a^	11.42^a^	12.80^a^	0.48	< 0.001
After treatment	5.51^b^	7.70^a^	5.48^b^	5.77^b^	5.48^b^	0.17	< 0.001
PCV%							
Before treatment	31.34^b^	32.92^a^	32.99^a^	31.87^a^	31.94^a^	0.15	0.008
After treatment	31.02^b^	32.10^a^	31.04^b^	31.67^ab^	30.74^b^	0.16	0.004
Hb g/dL							
Before treatment	11.75	11.82	11.80	11.76	11.72	0.08	0.996
After treatment	11.89	11.64	11.82	11.79	11.69	0.08	0.868

Mean values and ±SEM of red blood cells (RBCs), white blood cells (WBCs), packed cell volume (PCV%) and hemoglobin (Hb) of all experimental groups subjected to experimental induction of ruminal lactic acidosis. Groups (*n* = 9/group, three replicate pens). Different letters in the same experimental period indicate the differences between groups observed (*p* ≤ 0.005). Control (healthy lambs without induction of ruminal acidosis) and four ruminal acidosis (RA) induced groups; RA (lambs afflicted by experimentally induced RA without treatment), RA/MEOs (lambs afflicted by experimentally induced RA and treated with microencapsulated essential oils), RA/Marine Cal-Algae lambs afflicted by experimentally induced RA and treated with marine calcareous algae), and RA/MEOs +Marine Cal-Algae (which include lambs afflicted by experimentally induced RA and treated with MEOs +Marine Cal-Algae).

## 3 Results

### 3.1 Vital parameters of experimentally induced RA-challenged lambs in response to MEOs+Marine Cal-Algae

The clinical examination of acidosis-challenged lambs before and after treatment is depicted in Table 2. Regarding the main effect of time, rectal temperature rose progressively from 38.14 °C before treatment to 38.44 °C after 3 days and 39.08 °C after 6 days (*p* < 0.001). In contrast, pulse rate (beats/min), respiratory rate (breaths/min), skin turgor time, and capillary refill time (s) declined significantly over time. In contrast, rumen motility increased from 1.37 to 2.07 to 3.49 contractions/3 min (*p* < 0.001). Concerning the effect of treatment, the untreated RA group departed maximum from the healthy control, showing the lowest temperature (38.04 vs. 39.16 °C) and rumen motility (1.17 vs. 3.89/3 min) together with the highest pulse rate (112.02 vs. 82.11 beats/min), respiratory rate (58.92 vs. 34.91 breaths/min), skin turgor time (3.94 vs. 1.00 s) and capillary refill time (2.83 vs. 1.00 s). All three supplemented groups improved these variables relative to RA, and the combined RA/MEOs + Marine Cal-Algae group was consistently the closest to control, recording the highest temperature and the lowest pulse rate, respiratory rate and skin turgor time among the treated groups (*p* < 0.001). Regarding the time × treatment interaction, before treatment the four acidosis groups were statistically indistinguishable from one another and differed markedly from control across every parameter. By day 6, the combined treatment had largely normalized the clinical picture; temperature, pulse rate, respiratory rate, rumen motility, skin turgor and capillary refill were comparable to the control group. Meanwhile, the untreated RA group remained clinically abnormal when compared to the control group.

**Table 2:** Efficacy of combined therapeutic aids comprising microencapsulated essential oils and Marine Cal-Algae on inflammatory attributes in lambs afflicted by experimentally induced ruminal acidosis.

Item	Lysozyme (μg/mL)	MPO (U/mL)	NO (μmol/L)	CRP (μg/mL)	IL-6 (pg/mL)
Effect of time					
Before RA treatment	180.25	29.60^a^	5.35^a^	5.02^a^	111.63
After 3 days of RA treatment	176.72	28.60^b^	4.62^b^	4.47^b^	112.51
After 6 days of RA treatment	177.91	29.81^a^	4.01^c^	4.42^b^	102.50
Effect of treatment					
Control	176.62^b^	32.27^a^	3.44^e^	2.32^d^	71.38^c^
RA	128.23^c^	25.51^c^	6.71^a^	7.80^a^	160.07^a^
RA/MEOs	193.83^a^	29.89^b^	4.83^b^	4.96^b^	108.24^b^
RA/Marine Cal-Algae	191.99^a^	29.49^b^	4.45^c^	4.23^c^	104.37^b^
RA/MEOs + Marine Cal-Algae	200.80^a^	29.52^b^	3.87^d^	3.88^c^	100.34^b^
Interaction: time × treatment					
Before treatment					
Control	175.25^b^	32.15^a^	3.45^b^	2.38^b^	70.98^b^
RA	180.57^a^	28.64^b^	5.67^a^	5.31^a^	120.89^a^
RA/MEOs	182.46^a^	29.37^b^	6.07^a^	5.90^a^	123.74^a^
RA/Marine Cal-Algae	180.31^a^	28.19^b^	5.88^a^	5.80^a^	121.86^a^
RA/MEOs + Marine Cal-Algae	182.66^a^	29.64^b^	5.69^a^	5.73^a^	120.69^a^
After 3 days of treatment					
Control	176.93^b^	32.00^a^	3.61^e^	2.45^e^	71.86^c^
RA	104.33^c^	25.30^d^	6.38^a^	7.77^a^	179.10^a^
RA/MEOs	198.73^a^	29.07^b^	4.80^b^	4.61^b^	108.08^b^
RA/Marine Cal-Algae -	196.02^a^	29.13^b^	4.24^c^	4.00^c^	103.22^b^
RA/MEOs + Marine Cal-Algae	207.60^a^	27.51^c^	4.07^d^	3.51^d^	100.30^b^
After 6 days of treatment					
Control	177.68^b^	32.67^a^	3.26^c^	2.12^c^	71.30^d^
RA	99.78^c^	22.60^c^	8.09^a^	10.33^a^	180.22^a^
RA/MEOs	200.30^a^	31.24^b^	3.62^b^	4.38^b^	92.90^b^
RA/Marine Cal-Algae -	199.63^a^	31.15^b^	3.22^c^	2.89^c^	88.04^b^
RA/MEOs + Marine Cal-Algae	212.15^a^	31.40^b^	1.84^d^	2.40^c^	80.04^c^
*p*-Value					
Effect of time	0.599	< 0.001	< 0.001	< 0.001	0.244
Effect of treatment	< 0.001	< 0.001	< 0.001	< 0.001	< 0.001
Interaction	< 0.001	< 0.001	< 0.001	< 0.001	< 0.001
SEM	5.59	0.32	0.10	0.18	10.34

Data are expressed as mean; SEM, standard error of the mean. Means of bearing different superscripts within a row differ significantly. Groups (*n* = 9/group, three replicate pens). Control (healthy lambs without induction of ruminal acidosis) and four ruminal acidosis (RA) induced groups; RA (lambs afflicted by experimentally induced RA without treatment), RA/MEOs (lambs afflicted by experimentally induced RA and treated with microencapsulated essential oils), RA/Marine Cal-Algae lambs afflicted by experimentally induced RA and treated with marine calcareous algae), and RA/MEOs +Marine Cal-Algae (which include lambs afflicted by experimentally induced RA and treated with MEOs +Marine Cal-Algae).

MPO, myeloperoxidase; NO, nitric oxide; CRP, C-reactive protein; IL-6, interlukin-6.

### 3.2 Hematological and blood pH examination of experimentally induced RA challenged lambs in response to MEOs+Marine Cal-Algae

#### 3.2.1 Hematological indices

The hematological indices of RA-challenged lambs before and after treatment were presented in Table 3. The hematological indices of RA-challenged lambs increased significantly (*p* ≤ 0.05) in WBCs and PCV% values in all groups before treatment compared to the control group. Lambs received MEOs alone or a therapeutic combination of MEOs+Marine Cal-Algae, which showed a significant decrease (*p* ≤ 0.05) in WBCs and PCV% values after treatment. In comparison to the control group, there were no significant differences in RBCs and Hb values in RA challenges before and after treatment trials. The effectiveness of therapeutic regimes was assessed by monitoring all of the aforementioned alterations and bringing them all back to normal after intervention.

**Table 3:** Efficacy of combined therapeutic aids comprising microencapsulated essential oils and Marine Cal-Algae on ruminal parameters of lambs afflicted by induced ruminal lactic acidosis (mixed-model repeated-measures analysis).

Item	Total volatile fatty acids (mmol/L)	Lactic acid (mmol/L)	Acetic acid (mmol/L)	Propionic acid (mmol/L)	Butyric acid (mmol/L)	Acetate: Propionate ratio
Effect of treatment						
Control	80.66 ± 0.57^e^	53.73 ± 0.50^c^	52.35 ± 0.71^a^	14.51 ± 0.17^d^	7.98 ± 0.06^d^	3.62 ± 0.05^a^
RA	119.46 ± 0.21^a^	86.08 ± 0.65^a^	40.56 ± 0.45^d^	22.58 ± 0.20^a^	11.70 ± 0.20^a^	1.83 ± 0.02^d^
RA/MEOs	86.03 ± 0.45^c^	56.93 ± 0.51^b^	48.33 ± 0.15^c^	17.69 ± 0.33^b^	9.75 ± 0.23^c^	2.75 ± 0.06^c^
RA/Marine Cal-Algae	89.83 ± 0.84^b^	58.92 ± 0.50^b^	47.03 ± 0.13^c^	18.12 ± 0.18^b^	10.67 ± 0.10^b^	2.60 ± 0.03^c^
RA/MEOs + Marine Cal-Algae	83.53 ± 0.40^d^	54.50 ± 0.45^c^	50.27 ± 0.08^b^	16.18 ± 0.08^c^	10.24 ± 0.19^b^	3.12 ± 0.02^b^
Time (day)						
D1	93.05 ± 2.55^a^	64.28 ± 1.95^a^	45.99 ± 0.80^b^	16.79 ± 0.31^b^	9.96 ± 0.16^a^	2.78 ± 0.09^a^
D2	91.08 ± 2.67^b^	63.93 ± 2.06^a^	46.48 ± 0.71^b^	17.80 ± 0.38^a^	10.10 ± 0.26^a^	2.67 ± 0.10^b^
D3	93.05 ± 2.57^a^	62.92 ± 2.15^b^	47.76 ± 0.97^a^	17.95 ± 0.47^a^	10.03 ± 0.28^a^	2.75 ± 0.12^a^
D4	93.04 ± 2.61^a^	61.65 ± 2.32^c^	48.89 ± 0.55^a^	17.53 ± 0.54^a^	9.77 ± 0.23^a^	2.89 ± 0.12^a^
D5	90.75 ± 2.67^b^	60.35 ± 2.51^d^	48.26 ± 1.11^a^	18.19 ± 0.76^a^	10.26 ± 0.29^a^	2.82 ± 0.14^a^
D6	90.45 ± 2.87^b^	59.04 ± 2.74^e^	48.87 ± 1.04^a^	18.63 ± 0.82^a^	10.28 ± 0.36^a^	2.80 ± 0.14^a^
*p*-Value						
Effect of treatment	< 0.001	< 0.001	< 0.001	< 0.001	< 0.001	< 0.001
Effect of time	< 0.001	< 0.001	0.004	0.020	0.169	0.091
Effect of treatment × time	< 0.001	< 0.001	< 0.001	< 0.001	< 0.001	< 0.001

Data are expressed as mean; SE refers to (standard error). Means of bearing different superscripts within a row differ significantly. Groups (n = 9/group, three replicate pens). Control (healthy lambs without induction of ruminal acidosis) and four ruminal acidosis (RA) induced groups; RA (lambs afflicted by experimentally induced RA without treatment), RA/MEOs (lambs afflicted by experimentally induced RA and treated with microencapsulated essential oils), RA/Marine Cal-Algae lambs afflicted by experimentally induced RA and treated with marine calcareous algae), and RA/MEOs +Marine Cal-Algae (which include lambs afflicted by experimentally induced RA and treated with MEOs +Marine Cal-Algae).

### 3.3 Behavioral adaptation to RA induction in response to MEOs+Marine Cal-Algae

**Figure 2:**
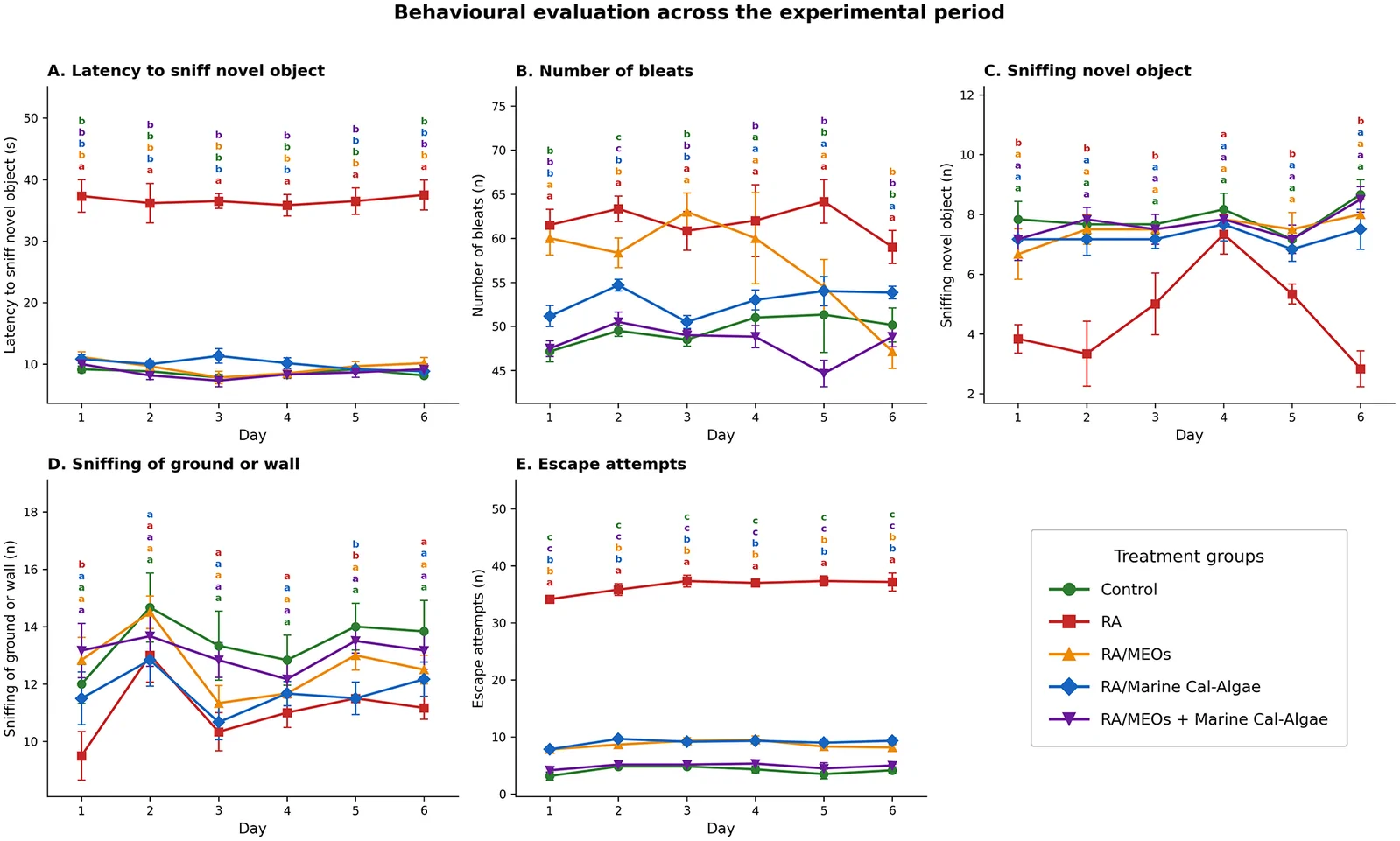
Ethograms of lambs' behavior during the open field test are afflicted by experimentally induced ruminal acidosis in response to combined therapeutic aids comprising microencapsulated essential oils and Marine Cal-Algae. Groups (*n* = 9/group, three replicate pens). Mean values (*p* ≤ 0.005) and standard error of means (±SEM) of escape attempts **(A)**, number of bleats **(B)**, sniffing novel object **(C)**, sniffing of the ground or wall **(D)**, and latency to sniff the novel object **(E)** of RA challenges before and after treatment. Control (healthy lambs without induction of ruminal acidosis) and four ruminal acidosis (RA) induced groups: RA (lambs afflicted by experimentally induced RA without treatment), RA/MEOs (lambs afflicted by experimentally induced RA and treated with microencapsulated essential oils), RA/Marine Cal-Algae lambs afflicted by experimentally induced RA and treated with marine calcareous algae), and RA/MEOs +Marine Cal-Algae (which include lambs afflicted by experimentally induced RA and treated with MEOs +Marine Cal-Algae).

Figure 2 shows ethograms of the sheep behaviors seen during the open field test. Following RA induction, all groups exhibited distinct nervous behavioral changes throughout the evaluation of the sheep's behavioral status during the study period. Thus, the connection between these various alterations in nerve behavior and experimental treatments was investigated. Regarding the open field test results in the current study, the frequencies of escape attempts (Figure 2A) and the number of beats (Figure 2B) in RA challenges before medical therapy showed notable significant elevations (*p* ≤ 0.05), while the frequencies of sniffing (Figure 2C) the novel object and the ground or wall (Figure 2D) showed notable significant drops (*p* ≤ 0.05) when compared to the control group. Meanwhile, the latency to sniff novel objects (Figure 2E) increased dramatically (*p* ≤ 0.05) prior to therapy, but it significantly decreased after treatment. These results indicated a relationship between ruminant physiological and behavioral responses that was significantly influenced by the therapeutic regimens administered to treated groups. These previously described changes in animal behavior following acidosis may be altered in treated groups at the end of 6 days of examination, particularly in animals fortified with MEOs+Marine Cal-Algae additive, and their parameters reverted to normal following intervention.

### 3.4 Serum inflammatory markers of RA-induced lambs in response to MEOs+Marine Cal-Algae

The serum inflammatory data are depicted in Table 4. Regarding the effect of time, nitric oxide and CRP fell steadily throughout the study (NO: 5.35 → 4.62 → 4.01 μmol/L; CRP: 5.02 → 4.47 → 4.42 μg/mL), while MPO dipped at day 3 (28.60 U/mL) before returning to its baseline value by day 6 (29.81 U/mL). Concerning the effect of treatment, untreated RA animals had the lowest lysozyme and MPO but the highest NO, CRP, and IL-6, reflecting an unresolved inflammatory state. All supplemented groups raised lysozyme above both the control and RA (193.83–200.80 μg/mL) groups and lowered the pro-inflammatory markers. The combined RA/MEOs + Marine Cal-Algae group produced the highest lysozyme and the lowest NO, with CRP and IL-6 reduced to near-control values (*p* < 0.001). Regarding the time × treatment interaction effect, group differences widened with time. By day 6, the untreated RA group displayed the most severe inflammatory profile (lysozyme: 99.78 μg/mL; NO: 8.09 μmol/L; CRP: 10.33 μg/mL; IL-6: 180.22 pg/mL), whereas the combined treatment reached the highest lysozyme (212.15 μg/mL) and the lowest NO (1.84 μmol/L), and IL-6 (80.04 pg/mL).

**Table 4:** Efficacy of combined therapeutic aids comprising microencapsulated essential oils and Marine Cal-Algae on remedial regimens of lambs afflicted by experimentally induced ruminal acidosis.

Group	Time in day					
	First day	Second day	Third day	Fourth day	Fifth day	Sixth day
Control	–	–	–	–	–	–
RA	0 (0%)	0 (0%)	0 (0%)	1 (11.11%)	3 (33.33%)	4 (44.44%)
RA/MEOs	0 (0%)	1 (11.11%)	3 (33.33%)	4 (44.44%)	6 (66.67%)	8 (88.89%)
RA/Marine Cal-Algae	0 (0%)	2 (22.22%)	4 (44.44%)	6 (66.67%)	7 (77.78%)	9 (100%)
RA/MEOs +Marine Cal-Algae	1 (11.11%)	3 (33.33%)	5 (55.55%)	6 (66.67%)	9 (100%)	9 (100%)

Data are expressed as mean; SEM, standard error of the mean. Means of bearing different superscripts within a row differ significantly. Groups (n = 9/group, three replicate pens). Control (healthy lambs without induction of ruminal acidosis) and four ruminal acidosis (RA) induced groups; RA (lambs afflicted by experimentally induced RA without treatment), RA/MEOs (lambs afflicted by experimentally induced RA and treated with microencapsulated essential oils), RA/Marine Cal-Algae lambs afflicted by experimentally induced RA and treated with marine calcareous algae), and RA/MEOs +Marine Cal-Algae (which include lambs afflicted by experimentally induced RA and treated with MEOs +Marine Cal-Algae).

### 3.5 Ruminal fluid analysis of RA-induced lambs in response to MEOs+Marine Cal-Algae remedies

#### 3.5.1 Ruminal liquor pH

**Figure 3:**
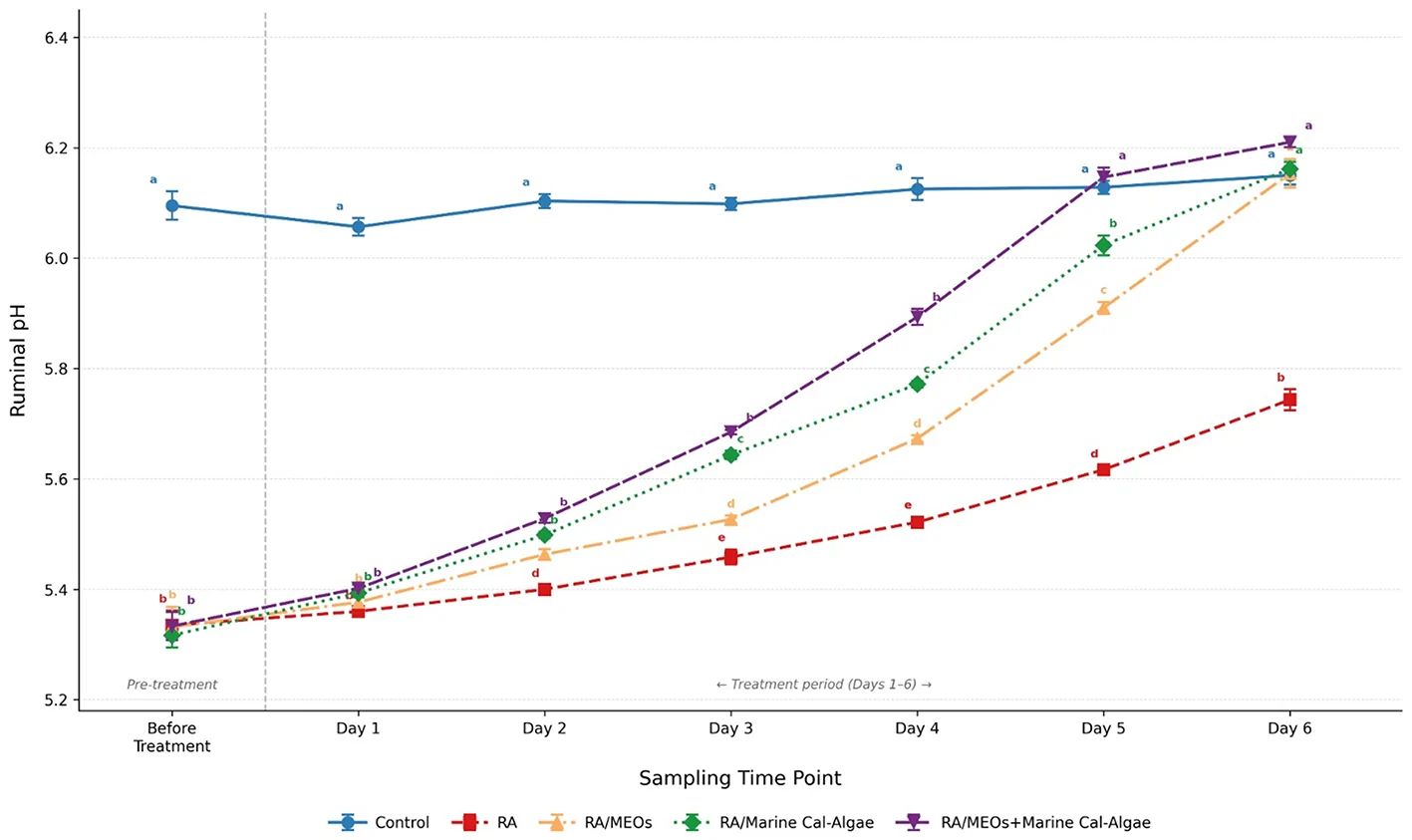
Ruminal pH from experimentally induced ruminal acidosis in response to combined therapeutic aids comprising microencapsulated essential oils and Marine Cal-Algae. Mean values before and during treatment. Control (healthy lambs without induction of ruminal acidosis) and four ruminal acidosis (RA) induced groups; RA (lambs afflicted by experimentally induced RA without treatment), RA/MEOs (lambs afflicted by experimentally induced RA and treated with microencapsulated essential oils), RA/Marine Cal-Algae lambs afflicted by experimentally induced RA and treated with marine calcareous algae), and RA/MEOs + Marine Cal-Algae (which include lambs afflicted by experimentally induced RA and treated with MEOs +Marine Cal-Algae).

The effect of the therapeutic regimens on ruminal pH across the pre-treatment sampling and the 6-day treatment period is presented in Figure 3. The data revealed significant differences among groups at every sampling point (*p* < 0.001). Prior to treatment, ruminal pH was significantly lower (*p* ≤ 0.05) in all four RA-challenged groups than in the control group, with no significant differences detected among the challenged groups, confirming a comparable baseline level of acidosis before treatment was applied. During the treatment period, ruminal pH increased progressively in all RA groups, but the magnitude and rate of increase differed significantly among them. From day 2 to 5, the RA/MEOs+Marine Cal-Algae group recorded the highest pH values among the challenged groups, followed by RA/Marine Cal-Algae, then RA/MEOs, with the untreated RA group remaining the lowest. By day 4, ruminal pH in the RA/MEOs+Marine Cal-Algae group had risen to 5.89 and in the RA/Marine Cal-Algae group to 5.77, compared with 5.67 in RA/MEOs and 5.52 in the untreated RA group. By day 6, ruminal pH in the RA/MEOs, RA/Marine Cal-Algae, and RA/MEOs+Marine Cal-Algae groups was statistically comparable to the control group (*p* ≤ 0.05). In contrast, the untreated RA group remained significantly lower. Across the whole period, the RA/MEOs+Marine Cal-Algae group showed the most rapid restoration of ruminal pH toward control values, reaching parity with the healthy group earliest among the treated groups.

#### 3.5.2 Ruminal lactic and total volatile fatty acids levels

**Figure 4:**
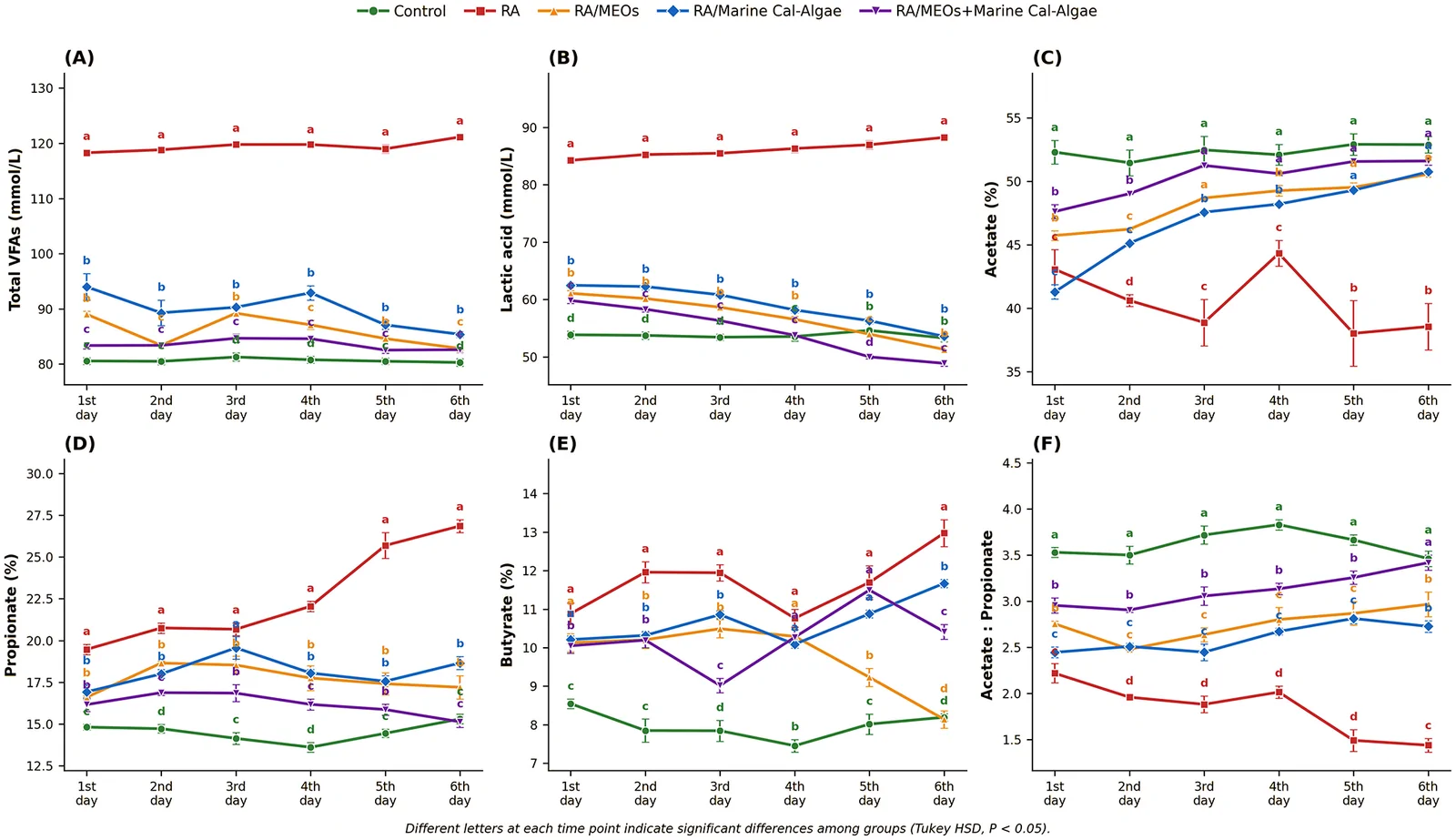
Ruminal volatile fatty acids from experimentally induced ruminal acidosis in response to combined therapeutic aids comprising microencapsulated essential oils and Marine Cal-Algae. Mean values before and during treatment. Groups (*n* = 9/group, three replicate pens). Mean values (*p* ≤ 0.005) and standard error of means (±SEM). Control (healthy lambs without induction of ruminal acidosis) and four ruminal acidosis (RA) induced groups; RA (lambs afflicted by experimentally induced RA without treatment), RA/MEOs (lambs afflicted by experimentally induced RA and treated with treatment microencapsulated essential oils), RA/Marine Cal-Algae lambs afflicted by experimentally induced RA and treated with marine calcareous algae), and RA/MEOs +Marine Cal-Algae (which include lambs afflicted by experimentally induced RA and were treated with MEOs +Marine Cal-Algae). **(A)** Total volatile fatty acids (VFAs), **(B)** lactic acid, **(C)** acetate, **(D)** propionate, **(E)** butyrate, **(F)** acetate: propionate.

The results of lactic and total volatile fatty acids analysis in response to MEOs+Marine Cal-Algae were presented in Figure 4. Ruminal lactic and total volatile fatty acids: lactic acid and TVFA showed significant increases (*p* ≤ 0.05) in challenged groups at the first day of treatment—RA (80.07 and 112.67 mmol/L), MEOs (62.47 and 93.99 mmol/L), Marine Cal-Algae (61.06 and 89.03 mmol/L), and MEOs+Marine Cal-Algae (59.81 and 83.36 mmol/L). After treatment, their levels decreased significantly (*p* ≤ 0.05): MEOs (53.55 and 85.39 mmol/L), Marine Cal-Algae (51.27 and 83.39 mmol/L), and MEOs+Marine Cal-Algae (48.86 and 82.60 mmol/L) by day 6.

Acidosis depressed acetic acid below control values, whereas propionic and butyric acids were elevated (*p* ≤ 0.05) relative to control values. After treatment, acetic acid was restored toward control values in all treated groups (50.74 in MEOs, 50.55 in Marine Cal-Algae, and 51.60 mmol/L in MEOs+Marine Cal-Algae by day 6). Lambs given MEOs+Marine Cal-Algae showed decreases (*p* ≤ 0.05) in propionic and butyric acids by day 6 (MEOs 18.65 and 11.66; Marine Cal-Algae 17.20 and 8.38; MEOs+Marine Cal-Algae 15.13 and 8.34 mmol/L). On the first day of treatment, the acetate-to-propionate ratio showed a notable reduction (*p* ≤ 0.05) in all RA-challenged groups compared to the control healthy one (2.41, 2.44, 2.75, and 2.95 in RA-induced, MEOs, algae, and MEOs+Marine Cal-Algae groups, respectively).

Additionally, treatment significantly affected all six ruminal fermentation parameters, and a significant treatment × time interaction was detected for every parameter (*p* < 0.001; Table 5). Averaged over the 6 days, untreated RA lambs recorded the highest total volatile fatty acid (119.46 mmol/L), lactic acid (86.08 mmol/L), propionic acid (22.58 mmol/L) and butyric acid (11.70 mmol/L) concentrations, together with the lowest acetic acid concentration (40.56 mmol/L) and acetate:propionate ratio (1.83), each differing significantly from the control group. For total VFA, all five groups differed significantly from one another, decreasing from RA (119.46) to RA/Marine Cal-Algae (89.83), RA/MEOs (86.03), RA/MEOs+Marine Cal-Algae (83.53), and control (80.66 mmol/L) groups. The combined RA/MEOs+Marine Cal-Algae regimen produced values nearest to control for the majority of the parameters; as lactic acid concentration (54.50 mmol/L) did not differ from the control (53.73 mmol/L) group, and it returned the highest acetic acid concentration (50.27 mmol/L) and acetate:propionate ratio (3.12) among the acidotic groups. A significant effect of time was observed for total VFA and lactic acid (*p* < 0.001; Table 5), acetic acid (*p* = 0.004) and propionic acid (*p* = 0.020). Lactic acid declined progressively over the treatment period, from 64.28 mmol/L on day 1 to 59.04 mmol/L on day 6, whereas acetic and propionic acids increased from their lowest values on day 1. Butyric acid and the acetate:propionate ratio were not significantly affected by time (*p* = 0.169 and *p* = 0.091, respectively).

**Table 5:** Efficacy of combined therapeutic aids comprising microencapsulated essential oils and Marine Cal-Algae on vital health parameters in lambs afflicted by experimentally induced ruminal acidosis.

Item	Temperature (°C)	Pulse rate/min	Respiratory rate/min	Rumen motility/3 min	Skin turgor/s	Capillary refill/s
Effect of time						
Before RA treatment	38.14^c^	112.96^a^	59.98^a^	1.37^c^	4.00^a^	3.20^a^
After 3 days of RA treatment	38.44^b^	108.06^b^	53.50^b^	2.07^b^	3.43^b^	3.23^b^
After 6 days of RA treatment	39.08^a^	84.50^c^	36.89^c^	3.49^a^	1.33^c^	1.20^c^
Effect of treatment						
Control	39.16^a^	82.11^e^	34.91^e^	3.89^a^	1.00^c^	1.00^c^
RA	38.04^d^	112.02^a^	58.92^a^	1.17^c^	3.94^a^	2.83^a^
RA/MEOs	38.45^c^	107.12^b^	54.17^b^	2.00^b^	3.44^b^	2.56^ab^
RA/Marine Cal-Algae	38.52^bc^	104.94^c^	52.56^c^	2.17^b^	3.17^b^	2.39^b^
RA/MEOs + Marine Cal-Algae	38.61^b^	103.00^d^	50.04^d^	2.33^b^	3.06^b^	2.28^b^
Interaction time × treatment						
Before treatment						
Control	39.15 ^a^	82.20 ^b^	35.00 ^b^	4.00^a^	1.00 ^b^	1.00^b^
RA	37.86 ^b^	120.40 ^a^	65.75 ^a^	0.67^b^	4.83 ^a^	3.67^b^
RA/MEOs	37.90 ^b^	121.20 ^a^	66.50 ^a^	0.83^b^	4.67 ^a^	3.83^b^
RA/Marine	37.89 ^b^	120.66 ^a^	66.85 ^a^	0.67^b^	4.67 ^a^	3.83^b^
RA/MEOs + Marine Cal-Algae	37.91 ^b^	120.33 ^a^	65.80 ^a^	0.67^b^	4.83 ^a^	3.67^b^
After 3 days of treatment						
Control	39.15 ^a^	82.14 ^e^	35.14 ^e^	3.86^a^	1.00 ^e^	1.00^d^
RA	37.80^e^	122.67^a^	66.83^a^	0.67^e^	4.67^a^	3.17^a^
RA/MEOs	38.13^d^	116.17^b^	60.00^b^	1.50^d^	4.33^b^	2.50^b^
RA/Marine Cal-Algae	38.45^c^	112.33^c^	55.67^c^	2.00^c^	3.83^c^	2.33^bc^
RA/MEOs + Marine Cal-Algae	38.66^b^	107.00^d^	49.83^d^	2.33^b^	3.33^d^	2.17^c^
After 6 days of treatment						
Control	39.18^a^	82.00 ^b^	34.60^b^	3.80^a^	1.00^c^	1.00^c^
RA	38.45^b^	93.00^a^	44.17^a^	2.17^b^	2.33^a^	1.67^a^
RA/MEOs	39.31a^a^	84.00^a^	36.00^b^	3.67^a^	1.33^b^	1.33^b^
RA/Marine Cal-Algae	39.23^a^	81.83^a^	35.17^b^	3.83^a^	1.00^c^	1.00^c^
RA/MEOs + Marine Cal-Algae	39.25^a^	81.67^a^	34.50^b^	4.00^a^	1.00^c^	1.00^c^
*p*-Value						
Effect of time	< 0.001	< 0.001	< 0.001	< 0.001	< 0.001	< 0.001
Effect of treatment	< 0.001	< 0.001	< 0.001	< 0.001	< 0.001	< 0.001
Interaction	< 0.001	< 0.001	< 0.001	< 0.001	< 0.001	< 0.001
SEM	0.15	1.96	1.49	0.46	0.46	0.42

Data are expressed as mean; SEM, standard error of the mean. Groups (*n* = 9/group, three replicate pens). Means of bearing different superscripts within a row differ significantly. Temperature °C, pulse rate/min, respiratory rate/min, Rumen motility/3-min, Skin turgor/second, and Blood capillary refill time/second of all experimental groups subjected to experimental induction of sub-acute ruminal lactic acidosis. Different letters in the same experimental period indicate the differences between groups observed (*p* ≤ 0.005). Control (healthy lambs without induction of ruminal acidosis) and four ruminal acidosis (RA) induced groups: RA (lambs afflicted by experimentally induced RA without treatment), RA/MEOs (lambs afflicted by experimentally induced RA and treated with treatment microencapsulated essential oils), RA/Marine Cal-Algae lambs afflicted by experimentally induced RA and treated with marine calcareous algae), and RA/MEOs +Marine Cal-Algae (which include lambs afflicted by experimentally induced RA and treated with MEOs +Marine Cal-Algae).

#### 3.5.3 Protozoa activity

**Figure 5:**
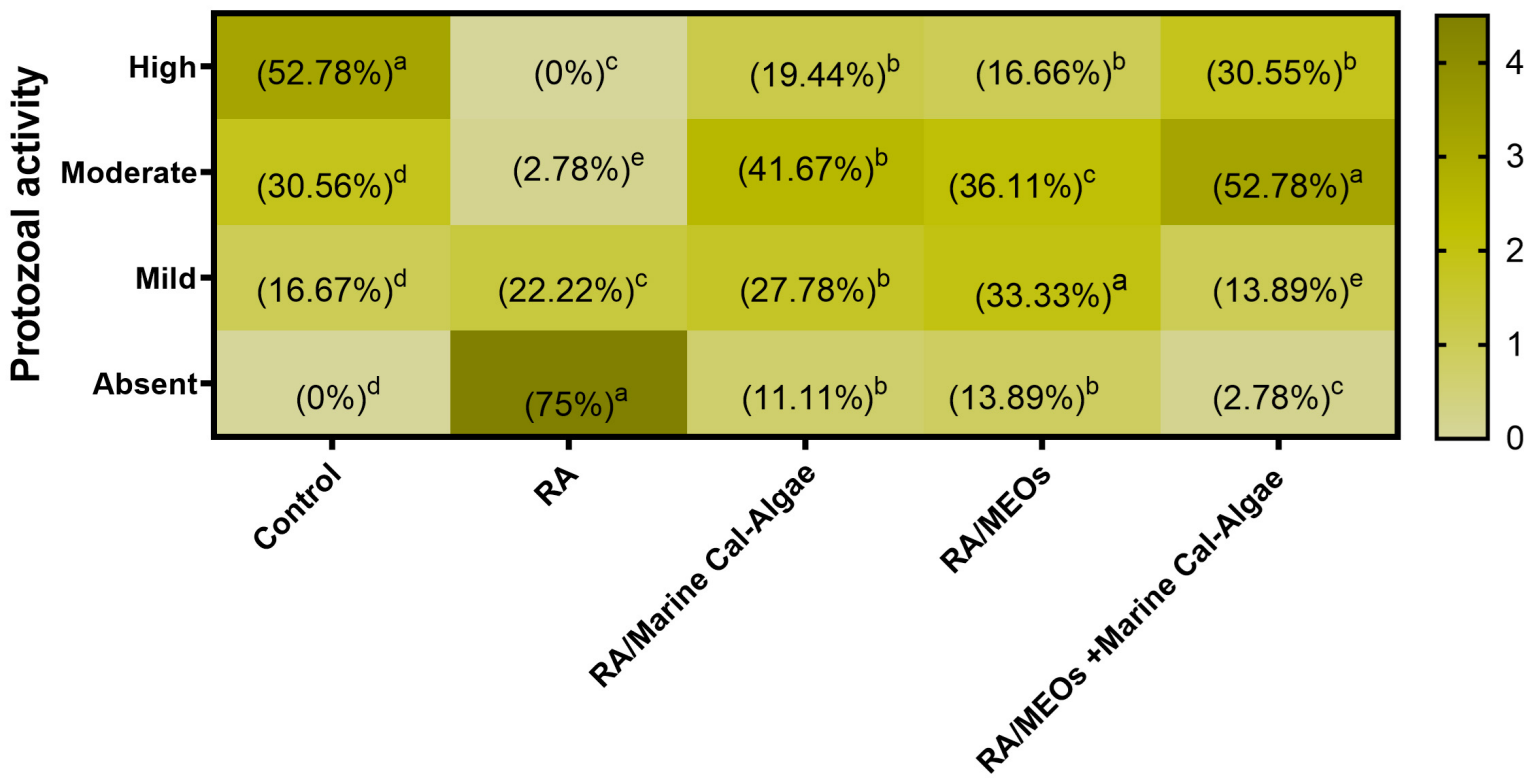
Heat map of ruminal protozoal activity of lambs afflicted by experimentally induced ruminal acidosis in response to combined therapeutic aids comprising microencapsulated essential oils and Marine Cal-Algae. Groups (*n* = 9/group, three replicate pens). Control (healthy lambs without induction of ruminal acidosis) and four ruminal acidosis (RA) induced groups: RA (lambs afflicted by experimentally induced RA without treatment), RA/MEOs (lambs afflicted by experimentally induced RA and treated with microencapsulated essential oils), RA/Marine Cal-Algae lambs afflicted by experimentally induced RA and treated with marine calcareous algae), and RA/MEOs +Marine Cal-Algae (which include lambs that afflicted by experimentally induced RA and treated with MEOs +Marine Cal-Algae).

The results of the protozoa activity test were summarized in Figure 5. Protozoa activity is an important criterion for metabolic acidosis diagnosis in sheep. Rather than an overall reduction, acidosis induction shifted the distribution of protozoal motility categories: post-acidosis, protozoa in the challenged groups were displaced from the high- and moderate-motility categories toward the low- and sporadic-motility categories, whereas the healthy control one retained predominantly high- and moderate-motility protozoa (*p* ≤ 0.05). Compared to the healthy group, protozoal motility was significantly improved (*p* ≤ 0.05) in RA challenges after medical therapy. The Marine Cal-Algae group showed greater improvement in protozoal activity than the MEOs group (moderate protozoal activity was 41.67 vs. 36.11%; high activity was 19.44 vs. 16.66%, respectively). The lambs that received MEOs+ Marine Cal-Algae mixture exhibited the highest improvement (*p* ≤ 0.05) in protozoa viability (the moderate protozoal activity was 52.78% and the high one was in 30.55% of treated lambs, respectively).

### 3.6 Remedial effectiveness of various therapeutic regimens

Clinical recovery from systemic acidosis was used to guarantee the remedial drugs' treatment accuracy, as indicated in Table 1. The therapeutic regimen was highly effective in correcting RA in rams in response to a combined treatment with both MEOs and Marine Cal-Algae. An obvious improvement was observed in the general health condition of affected animals post-treatment. When we evaluated the effectiveness of the therapeutic agents, MEOs+ Marine Cal-Algae was the most effective therapy on the fifth day post-therapy, followed by Marine Cal-Algae on the sixth day post-therapy. Then MEOs was placed last based on lambs' clinical recovery. Both remedial agents were able to treat the acidotic condition. Still, the combination of MEOs and calcareous marine algae in acidotic rams was the most effective choice to correct the detrimental effects of metabolic acidosis on lambs' health and performance.

## 4 Discussion

Sheep contribute substantially to agricultural productivity and are essential for the livelihood security of resource-limited pastoralists. Rumen acidosis is a predominantly management-driven condition, often arising from inadequate dietary practices, especially in intensively fed animals (39). Ruminal acidosis (RA) is a nutrition-related metabolic condition induced by the overconsumption of rapidly fermentable carbohydrates, frequently occurring in high-yielding dairy cattle and feedlot sheep (40). In the current study, the classic clinical signs of RA, including weakness, dullness, ruminal stasis, anorexia, pasty feces, dyspnea, teeth grinding, unwillingness to move, and tympany following rumen acidosis induction, were successfully induced in the challenged lambs; these results are consistent with those of Ribeiro et al. (41). The clinical signs resolved after treatment in all acidotic lambs when appropriate treatment with Marine Cal-Algae was used alone or in combination with MEOs. Accordingly, the detrimental impact on animal health and performance is eliminated when calcareous marine algae are used to treat lactic acidosis (42). Before receiving medical therapy, the clinical examination of this study demonstrated a discernible rise in skin turgor, capillary refill time, and respiratory and pulse rates. In contrast, body temperature and ruminal motility were decreased. All the findings from the current investigation were comparable to those from Abebaw al. (37). Increased blood carbon dioxide tension and decreased blood pH activated the medulla oblongata, raising the respiration rate above normal (43). Additionally, activation of the sympathetic nervous system due to metabolic acidosis caused an increase in pulse rate in RA-induced lambs (37). Moreover, fluid and electrolyte loss caused the current study's reported drop in body temperature (44). Similar results were found in acidotic lambs with increased skin turgor and capillary refill time (45), because of diarrhea and dehydration recorded (46). Deactivation of the sympathomimetic ganglion caused hypomotility or muscle atony of the rumen under grain loading (47). Ruminal motility and body temperature improved in RA-challenged lambs supplemented with MEOs and Marine Cal-Algae, in addition to a discernible drop in respiratory and pulse rates. Following treatment trials, there was a significant decrease in skin turgor and blood capillary refill time. Accordingly, essential oil mixtures manage ruminal acidosis by selectively inhibiting bacteria that produce lactic acid, thereby stabilizing ruminal pH and decreasing fermentation risks (14). Furthermore, rumen buffering with Marine Cal-Algae may increase fiber digestibility and acetate production, which might increase methane emissions, which plays a crucial role in preventing the accumulation of metabolic hydrogen in the rumen that would inhibit microbial activity (48). Moreover, essential oils from plants like oregano have been shown to promote beneficial microbial populations (*Ruminococcus flavefaciens, Ruminococcus albus*, and *Fibrobacter succinogenes*), making them effective natural alternatives to chemical additives (49). Research indicates that essential oils affect the functional dynamics of the rumen microbiome, often mimicking the action of ionophores by selectively inhibiting methanogenic microbes while promoting the growth of fiber-degrading bacteria (50). By reducing the accumulation of lactate, essential oils prevent the drastic drop in pH associated with ruminal acidosis, especially in high-concentrate diets (16). By limiting the rapid degradation of readily fermentable carbohydrates (starch) into lactate, EOs prevent the drastic drop in ruminal pH that characterizes RA (15). Certain oils, such as those found in thyme, clove, and cinnamon, can increase the proportion of propionate and decrease acetate, leading to more efficient energy metabolism (5) VFA Management. The antibacterial activity of the essential oil mixture and the buffering action of Marine Cal-Algae, which neutralize excess ruminal acids produced after acidosis, are responsible for the improvement in all vital signs, which is in line with the results of Hassan et al. (42). The antimicrobial efficacy of cinnamaldehyde, the primary non-phenolic phenylpropene in cinnamaldehyde (CIN), is attributed to the interaction of its carbonyl functional group with periplasmic proteins, ultimately leading to the inactivation of essential microbial enzymes (51).

Regarding open field test results, there were marked, significant elevations in frequencies of escape attempts and number of beats in RA challenges prior to medical therapy. In contrast, the frequencies of sniffing the novel object and sniffing the ground or wall exhibited a notable significant drop compared to the control group. Meanwhile, latency to sniff the novel object increased significantly in acidotic animals before treatment but decreased significantly following treatment. Accordingly, feeding motivation has been identified as an important determinant of exploratory behaviors in animals (52). Consequently, a lower acetate-to-propionate ratio, acidic ruminal pH, and the alterations in ruminal microbiota observed in acidotic rams (53) may be predisposing factors for diminishing feeding motivation or appetite, which is associated with a subsequent reduction in exploratory behaviors in rams (54). These changes are considered the main causes of physiological and behavioral responses of ruminants under metabolic acidosis stress; this explanation was last discussed by Qianrige et al. (26). Additionally, additive regimens in treated groups may significantly modulate physiological and behavioral responses in ruminants. Notably, all previously mentioned behavioral disturbances were corrected in treated groups, especially in animals fortified with Marine Cal-Algae additive either alone or in combination with MEOs, and their parameters returned to normal after intervention. The buffering action of Marine Cal-Algae on the ruminal environment could remove the harmful effect of RA on ruminal liquor parameters and improve animal health and viability, which is in line with Abd-El Razek et al. (21). Additionally, WBCs and PCV% values in the ongoing investigation of RA-challenged rams were significantly elevated prior to treatment. Still, RBCs and Hb values showed no remarkable changes in RA-challenged rams before or after treatment trials. Similar to Zein-Eldin et al. (55), an increase in WBCs and PCV was noted in acidotic sheep. Endotoxin generation is the cause of the rise in WBCs levels in ruminal acidosis; these findings were reported by Padmaja and Gopala (56). Dehydration and blood concentration from fluid and electrolyte loss due to severe diarrhea were the causes of elevated PCV in sheep with systemic acidosis (57). The aforementioned changes were attributed to notable dehydration, malnutrition, and associated immunosuppression (58). Pre- and post-therapy, RBC and Hb values did not exhibit any significant changes and remained within the reference ranges specified by Jackson et al. (59). These findings were consistent with those of Elmeligy et al. (60), as seen by declining WBCs and PCV% values; lambs given a dietary supplementation of Marine Cal-Algae feed additives with or without MEOs showed the most notable hematological reaction. The existing study's lysozyme and MPO activity results demonstrated a considerable drop in all acidotic groups prior to medication, but following therapeutic intervention, their values dramatically improved. Moreover, systemic inflammation caused by RA induction in lambs also increased the levels of NO, CRP, and IL-6 in the sera of afflicted lambs prior to therapy. RA destroys the rumen epithelial barrier, triggering an inflammatory reaction. In the current study, the group that received MEOs showed a significant immunostimulant effect, as supported by alleviation of the excessive inflammatory response following RA induction. Previous research reported that essential oils mainly derived from oregano and clove exhibit antimicrobial properties that can affect the microbial populations within the rumen, reduce dry matter ruminal degradability, leading to lower VFA production, and reduce systemic inflammation (14). In our study, the use of Marine Cal-Algae, either alone or in conjunction with MEOs, can reduce systemic inflammation in acidotic groups by raising lysozyme and MPO levels and lowering NO, CRP, and IL-6 levels. The buffering effect of Marine Cal-Algae on the rumen can improve health conditions and effectively treat acidosis by stabilizing rumen pH, neutralizing the production of excess volatile fatty acids, reducing endotoxin leakage into the bloodstream, correcting ruminal acidosis, and suppressing systemic inflammation and oxidative stress, which agrees with Loučka et al. (61). In the present study, lactic and total volatile fatty acid levels showed highly significant elevation in the acidotic group (pre-therapy). The acetate-to-propionate ratio also demonstrated a significant decrease in RA challenges before treatment. According to El-Katcha et al., metabolic acidosis tends to lower the levels of acetic acid while dramatically increasing the levels of propionic and butyric acids (62). Lactic acid builds up in the rumen and significantly lowers rumen pH in acidosis, increasing the production of TVFA. These results were further supported by earlier research by Shen et al. (63). Notably, a high-concentrate meal promotes microbial fermentation and produces VFAs that exceed the epithelium's capacity to absorb them, as noted by Mao and Wang (64). The addition of essential oils as a medical remedy results in a shift in VFA profiles, promoting the production of propionate more than acetate, consequently improving feed efficiency, as noted by Zhang et al. (65). In treated lambs, the integrated therapy using algae alone or mixed with MEOs significantly reduced lactic and TVFA values and increased the production of acetic acid in the rumen, which agreed with Sarmikasoglou and Faciola (66). The current study's statistical analysis of ruminal liquor pH data revealed a significant drop in RA-challenged groups following the onset of acidosis. When rumen acidosis occurs, the rumen wall's ability to absorb VFAs and lactic acid is significantly impaired, leading to a downward spiral in pH and hindered energy absorption. High-concentrate diets often promote stable foam formation, which creates a physical barrier that inhibits their absorption (64). When acidosis occurs, the rumen wall is unable to absorb VFAs and lactic acid energy molecules; these findings are attributed to reduced saliva output and a sharp drop in ruminal pH. Consequently, the accumulation of these acids causes rumenitis, which damages the rumen epithelial cells and disrupts their absorption and barrier functions, as reported by Fu et al. (2). Adding plant extracts to enhance fermentation could be used to treat RA, as previously mentioned by Loučka et al. (61). The findings demonstrated that algae-based feed additives and MEOs could considerably alleviate RA induced in rams. Clinical improvement was noted in the MEOs group because essential oils contain various bioactive compounds that modify rumen fermentation profiles by improving the growth of beneficial bacteria, diminishing other pathogens, enhancing nutrient utilization efficiency, and correcting acidic rumen pH (14). High mineral content in Marine Cal-Algae can improve rumen pH by increasing water intake, diluting acids, and decreasing propionate synthesis in the rumen, which can be considered an effective approach for correcting RA (21). The results of the current investigation revealed a highly significant decrease in protozoa motility in all experimental groups compared to the control group following acidosis. In line with our findings, Padmaja and Gopala (56) reported that the acidic pH of rumen liquor negatively impacted the concentration and motility of rumen protozoa. The observed reduction in protozoa motility after acidosis is consistent with the findings of Dagnaw Fenta et al. (39). Following medical treatment, protozoa motility improved, although the MEOs group's improvement was less than that of the Marine Cal-Algae group. Protozoa viability most significantly improved in the lambs fed with the MEOs+Marine Cal-Algae, which may be relatively close to the return of rumen pH to a normal level. Compared to their pretreatment state, the lambs' health and vitality were enhanced by dietary Marine Cal-Algae fortification, which reduced the adverse effects of lactic acidosis, in agreement with Kholif and Olafadehan (67).

In the current study, depending on lambs' recovery, all remedial agents, especially their combination (MEOs and Marine Cal-Algae), were able to correct lactic acidosis effectively. MEOS are recognized for their antimicrobial properties, which can shift the microbial population of the rumen, impact fermentation processes, and improve animal health and welfare (67). Marine Cal-Algae contains high levels of calcium and magnesium, which act as a buffer to neutralize excess volatile fatty acid production in the rumen resulting in compatibility (61). Its polymorphic structure allows for a slower release of these minerals compared to soluble buffers. MEOs can minimize the leakage of endotoxins from the rumen into the bloodstream. It can also inhibit inflammatory pathways and lower systemic inflammation. Therefore, the combination therapy of MEOs and Marine Cal-Algae constitutes the most powerful therapeutic agent for the treatment of acute lactic ruminal acidosis.

## 5 Conclusion

Ruminal acidosis is fundamentally a management disorder driven by the overconsumption of rapidly fermentable carbohydrates. The clinical, vital, ruminal, hematological, biochemical, and behavioral changes recorded here confirmed that the grain-overload protocol successfully induced ruminal (lactic) acidosis in lambs. Under the conditions of this study, microencapsulated essential oils (MEOs) and marine calcareous algae (CMA) effectively supported recovery, with the combined regimen being the most effective. Collectively, the treatments reduced VFA and lactic acid accumulation and attenuated inflammatory and behavioral responses, with the combined regimen producing the best overall outcomes. Although the combination was the most effective, its practical adoption on a larger scale should also take economic considerations into account in the future field studies. Additionally, gavage with wheat flour resulted in high ruminal lactate concentrations, indicating acute lactic ruminal acidosis rather than classical subacute ruminal acidosis; therefore, the therapeutic responses observed in this study should be further evaluated to investigate their efficacy against subacute ruminal acidosis, which occurs more frequently under field conditions.

## Data Availability

The data presented in this study are available in the article, and upon request from the corresponding author.
